# Impact of Age, Marital Status, Smoking, and Alcohol Consumption on Urinary and Sexual Function in Prostate Cancer Patients Treated With Radical Prostatectomy: A Prospective Cohort Study

**DOI:** 10.1016/j.urology.2025.07.048

**Published:** 2025-07-25

**Authors:** Christie Zhang, Andrew Harper, Kellie R Imm, Robert L Grubb, Eric H Kim, Graham A Colditz, Kathleen Y Wolin, Adam S Kibel, Lin Yang, Siobhan Sutcliffe, Saira Khan

**Affiliations:** 1 Western University, London, Ontario, Canada; University of British Columbia, Vancouver, British Columbia, Canada.; 2 Alberta Health Services, Edmonton, Alberta, Canada.; 3 Department of Preventive Medicine, Keck School of Medicine of University of Southern California, Los Angeles, CA.; 4 Department of Urology, Medical University of South Carolina, Charleston, SC.; 5 Department of Urology, University of Nevada-Reno, Reno, NV.; 6 Division of Public Health Sciences, Department of Surgery, Washington University in St. Louis School of Medicine, St. Louis, MO.; 7 Circea, Chicago, IL.; 8 Department of Urology, Mass General Brigham, Harvard School of Medicine, Boston, MA.; 9 Division of Public Health Sciences, Department of Surgery, Washington University in St. Louis School of Medicine, St. Louis, MO.

## Abstract

**OBJECTIVE:**

To examine the impact of age, marital status, smoking status, and alcohol use on urinary and sexual function up to 1 year post-radical prostatectomy (RP) among men with prostate cancer.

**METHODS:**

Participants were recruited from the Prostatectomy, Incontinence, and Erectile dysfunction study. Patient characteristics were assessed at baseline. Urinary and sexual function were assessed using the Expanded Prostate Cancer Index Composite-50 at baseline and 5 weeks, 6 months, and 12 months post-RP. Mean urinary and sexual function scores were calculated for each time point by patient characteristics.

**RESULTS:**

The cohort consisted of 311 and 286 men for urinary function and sexual function, respectively. *Urinary function*: At baseline, married men had significantly higher urinary function scores (*P* = .027). By 6 months post-RP, older (vs younger, *P* = .044) and unmarried men (vs married, *P* = .008) had significantly worse urinary function. By 12 months post-RP, these differences disappeared, with all groups returning to levels approaching baseline. *Sexual function:* At baseline, participants who were younger (*P* < .001), never-smokers (*P* < .001), and more frequent consumers of alcohol (*P* = .021) had significantly higher sexual function scores. Small improvements in sexual function occurred at 6 months post-RP. By 12 months post-RP, sexual function did not recover to baseline levels for any group and was significantly lower for older (*P* < .001), unmarried (*P* < .025), and ever-smoker participants (*P* = .002).

**CONCLUSION:**

Urinary function scores recover to levels approaching baseline by 12 months post-RP. Sexual function scores do not recover to baseline levels by 12 months post-RP for any group and are lower for participants who are older, unmarried, or smoke.

Prostate cancer is the most common cancer among men in the United States, with a 5-year relative survival of over 97%.^1^ This relative survival is partly attributable to highly effective definitive treatments for prostate cancer, including radical prostatectomy (RP) and radiation. Although these treatments are considered “curative,” they can cause significant adverse side effects that negatively impact men’s quality of life. In particular, 20% of men experience urinary incontinence after surgery and 66% of men experience sexual dysfunction.^2^ These treatment–related side effects can persist for several months after treatment and may never fully resolve. In the Prostatectomy, Incontinence, and Erectile function (PIE) study, a prospective cohort study of men treated with RP, we found that 41.8% and 69.0% of patients do not recover their urinary and sexual function, respectively, to baseline levels at 1-year post surgery, indicating that adverse-treatment–related side effects are both prevalent and persistent.^3^ This contributes to a higher incidence of treatment–related decisional regret among men receiving RP as compared to other treatments, with sexual function–related side effects driving these findings.^4^ Identifying modifiable and non-modifiable contributing factors for adverse-treatment–related effects post-RP is key in improving clinicians’ ability to counsel patients and potentially mitigate these side effects.

In general, older prostate cancer patients have poorer urinary and sexual function recovery following RP.^5,6^ Evidence also suggests that factors such as marital status and modifiable factors such as cigarette smoking and alcohol consumption may play a role in post-RP recovery. Married men generally have better prostate cancer outcomes throughout the cancer continuum; however, few studies have translated this association to post-RP recovery.^7,8^ With respect to cigarette smoking and drinking alcohol, surgical patients are generally advised to quit smoking^9^ and reduce their alcohol consumption^10^ to optimize surgical recovery, as both are associated with negative outcomes. However, such fundamental behavioral change can be stressful and limited studies have been conducted to support this practice for post-RP recovery.^11,12^

Given these gaps in knowledge, we aimed to examine the impact of age, marital status, cigarette smoking, and alcohol consumption on patterns of urinary and sexual recovery post-RP in the PIE longitudinal study. We hypothesize that men who are younger, married, non-smokers, or have limited alcohol use will experience a faster recovery of urinary and sexual function after treatment with RP.

## MATERIALS AND METHODS

### Study Population and Design

Patients with clinically localized prostate cancer who underwent RP at either Washington University School of Medicine or Brigham & Women’s Hospital between September 2011 and January 2014 were recruited into the PIE longitudinal study. Exclusion criteria included (1) previous treatment for prostate cancer, salvage therapy, radiation treatment to the pelvis (including bladder, rectum, or prostate), or major pelvic surgery (including penile implant or urinary sphincter); (2) known urethral stricture or colostomy; (3) chronic urinary catheterization; (4) non-English speaking, and (5) receipt of neo-adjuvant therapy or any additional prostate cancer–related therapies (eg, radiation or hormonal therapies) during the 1-year study follow-up. These exclusion criteria were applied during both recruitment and analyses.

After obtaining informed consent, questionnaires were administered to participants at 4 time points: before RP (baseline) and at 5 weeks, 6 months, and 12 months after surgery. The questionnaires covered a wide array of topics, including socio-demographic characteristics, cigarette smoking history, alcohol consumption level, and urinary and sexual function.^3^ The PIE study was approved by the review boards at Washington University School of Medicine and Brigham & Women’s Hospital.

### Main Exposures: Age, Marital Status, Smoking, Alcohol Consumption

Age, marital status, smoking, and alcohol consumption were assessed by self-report on the baseline questionnaire. Age was dichotomized at 65 years of age, consistent with the National Institute of Aging’s definition of older age. Marital status was defined as married or living with a partner vs all other categories. Smoking was defined as ever-smokers (including former and current) vs never-smokers. Former and current smokers were combined due to the small number of current smokers in our cohort. Alcohol consumption was categorized as never, monthly, weekly, and daily drinking. All exposures were measured at baseline.

### Main Outcomes: Urinary and Sexual Outcomes

Urinary and sexual outcomes were measured using the Expanded Prostate Cancer Index Composite-50 (EPIC-50) instrument.^13^ EPIC-50 is a prostate cancer–specific and patient-reported scale that measures quality of life. It is one of the most used tools in prostate cancer–specific research and was created from the validated University of California, Los Angeles Prostate Cancer Index.^13^ Here, we focused on urinary and sexual function as these are the most common treatment–related side effects following RP. The EPIC-50 was administered before RP (baseline) and at 5 weeks, 6 months, and 12 months after surgery.

### Statistical Analysis

For both urinary and sexual domains, a summary score was calculated and transformed linearly to a numerical scale ranging from 0–100, with lower numbers representing worse function.^13^ Participants’ summary scores were calculated at each of 4 time points: baseline, and 5 weeks, 6 months, and 12 months post-treatment. We calculated the mean and standard deviation of scores at each time point. Scores were stratified by our exposures of interest: age, marital status, smoking, and alcohol use. Significant differences in mean scores, for each exposure and at each time point, were examined using *t* tests for dichotomous exposures and analysis of variance for multi-category exposures. In a sensitivity analysis, we adjusted mean scores for age. A *P* value less than or equal to .05 was considered statistically significant.

## RESULTS

### Cohort Characteristics

Of the 426 participants recruited to the PIE study, we excluded 22 who received additional therapies during follow-up and 55 who did not complete surveys at 5 weeks, 6 months, and 12 months. This resulted in 349 eligible participants (86.4% of 404 participants who did not receive additional therapies), of whom 311 men provided information on urinary function and 286 men provided information on sexual function at all 3 time points and did not have missing information on the exposures of interest. Cohort characteristics are presented in Table 1. Over 90% of participants self-identified as White and mean age was 61 years. Most participants were married or living with a partner (> 85%), had never smoked (> 58%), and consumed alcohol weekly to daily (> 57%). Most patients (∼80%) received bilateral or unilateral nerve-sparing surgery.

### Urinary Function

**Figure 1.**
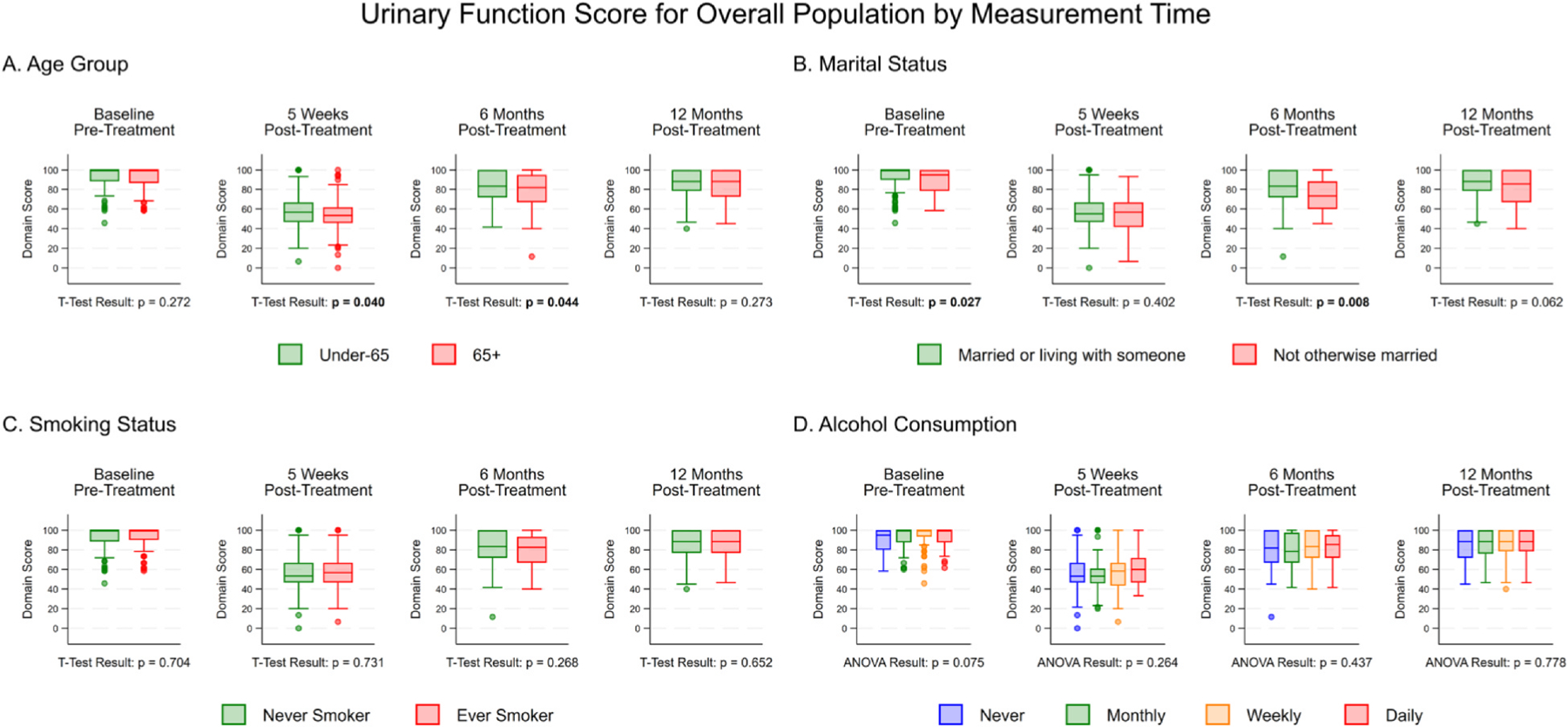
Box and whisker plot for mean urinary function score at 4 time points, baseline, 5 weeks post-treatment, 6 months post-treatment, and 12 months post-treatment, by **(A)** age, **(B)** marital status, **(C)** smoking status, and **(D)** alcohol consumption. Significant findings (P ≤.05) are bolded. At 6 months post-treatment, urinary function scores began to improve for all men, but older and unmarried men experienced slower recovery. By 12 months post-treatment, there were no significant differences by group, and scores approached, but did not reach baseline levels. ANOVA, analysis of variance.

Urinary function scores at each of the 4 time points are presented in Figure 1 by age (A), marital status (B), smoking status (C), and alcohol consumption (D). Unadjusted and age-adjusted mean scores are presented in Supplemental Tables 1 and 2, respectively. At baseline, there were no significant differences in mean urinary function by age, smoking status, or alcohol consumption. However, married men or men living with partner had significantly higher urinary function scores at baseline (*P* = .027). At 5 weeks post-treatment, mean scores decreased for all groups, but were significantly lower for older men (≥65 years) vs younger men (< 65 years, *P* = .04). At 6 months post-treatment, urinary function began to improve for all men. However, older (vs younger, *P* = .044) and unmarried (vs married, *P* = .008) men continued to have significantly lower urinary scores, suggesting slower improvement in older and unmarried men. By 12 months post-treatment, all groups experienced a similar improvement in urinary function, with scores approaching, but not reaching, baseline scores. There were no significant differences observed in urinary function between men by age, marital status, smoking status, and alcohol frequency. Mean scores by marital status, smoking status, and alcohol consumption were consistent after age-adjustment.

### Sexual Function

**Figure 2.**
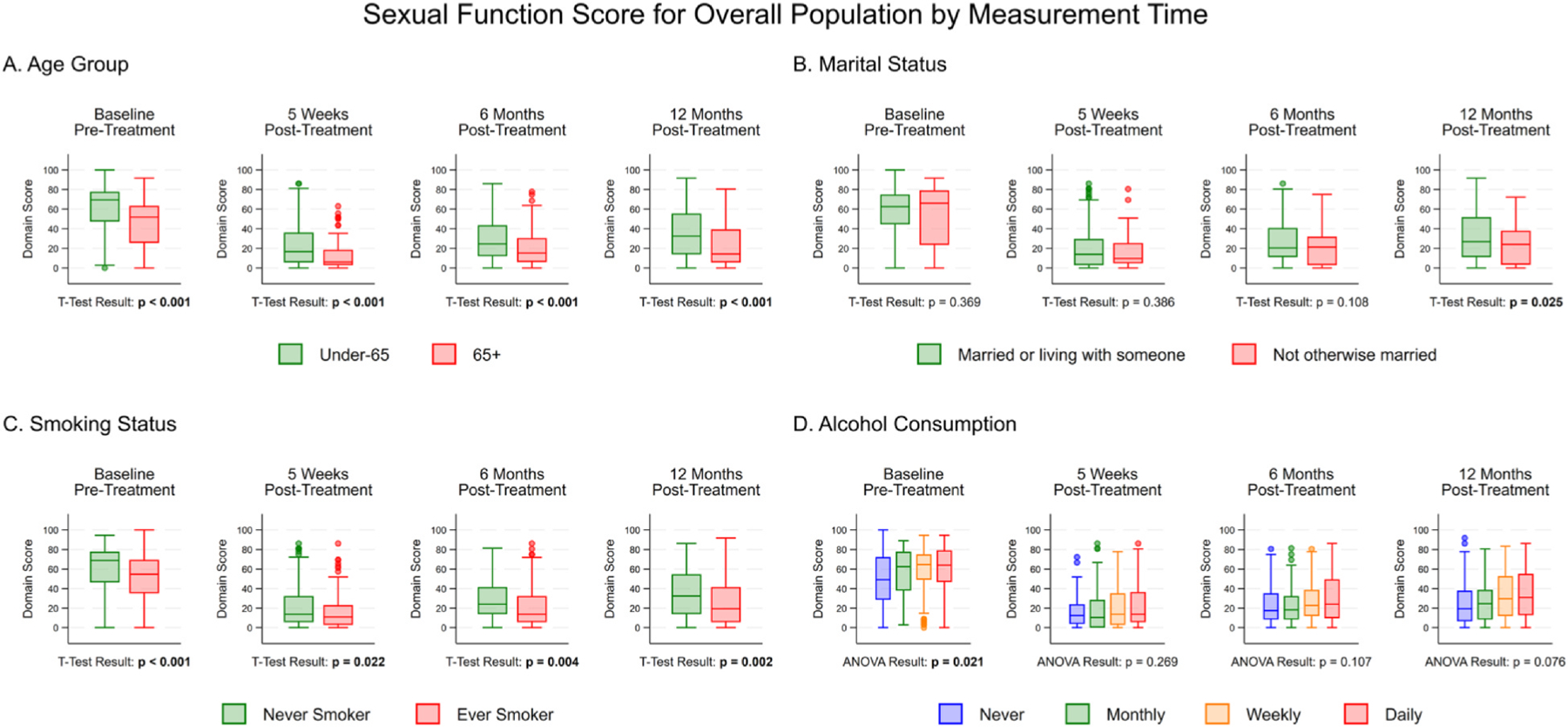
Box and whisker plot for mean sexual function score at 4 time points, baseline, 5 weeks post-treatment, 6 months post-treatment, and 12 months post-treatment, by **(A)** age, **(B)** marital status, **(C)** smoking status, and **(D)** alcohol consumption. Significant findings (P ≤.05) are bolded. At 6 months post-treatment, all groups had lower sexual function scores than baseline, with older men and ever-smokers having significantly lower scores. At 12 months post-treatment, scores improved, but did not recover to baseline levels for any group, with older, unmarried participants, and ever-smokers having significantly lower scores. ANOVA, analysis of variance.

Sexual function scores at each of the 4 time points are presented in Figure 2 by age (A), marital status (B), smoking status (C), and alcohol consumption (D). Unadjusted and age-adjusted mean scores are presented in Supplemental Tables 3 and 4, respectively. At baseline, participants who were older than 65 years of age (*P* < .001), ever-smokers (*P* < .001), and less frequent consumers of alcohol (*P* = .021) had significantly lower sexual function scores. At 5 weeks post surgery, all men experienced a decrease in sexual function, with older men (*P* < .001) and smokers (.022) having significantly lower sexual function scores. No significant differences were observed by marital status and alcohol consumption. At 6 months post-treatment, there was a small improvement in sexual function for all men, but scores remained at clinically relevant lower levels^14^ than at baseline (baseline mean scores, range: 48–62 across exposures; 6 months mean scores, range: 23–31 across exposures; Supplemental Table 3). The mean drop in score from baseline to 6 months was 30 points across exposures. At 6 months post-treatment, older men (≥65 years, *P* < .001) and smokers (*P* = .004) continued to have significantly lower sexual function scores. At 12 months post-treatment, there were additional improvements in sexual function; however, for all exposure categories, function did not recover to baseline levels (12 months mean scores, range: 23–35 across exposures; mean drop in score from baseline: 26 points across exposures). By 12 months post-treatment, sexual function scores were significantly lower for older (*P* < .001), unmarried participants (*P* < .025), and ever-smokers (*P* = .002).

## DISCUSSION

### Summary

In our study, we observed that most participants started with similar levels of urinary function at baseline and then followed a similar trajectory of decline and recovery. Patients who were married (vs not married) had significantly higher baseline scores and older men (vs younger men) had lower scores at 5 weeks and 6 months post-treatment, but by 12 months post-RP there were no differences across exposure groups. In comparison, for sexual function outcomes, patients who were younger, never-smokers, and more frequent consumers of alcohol started with higher baseline scores. At 12 months post-treatment, significant differences persisted across many of these exposure groups, with significantly higher scores among younger men, married men, and never-smokers. For all men, sexual function scores did not recover to baseline levels at 12 months post-treatment.

### Urinary Function

In the current literature, older age has been commonly identified as a risk factor for post-RP urinary incontinence. Two systematic reviews concluded that increasing age is a significant predictor of urinary incontinence in patients who undergo RP and a relevant factor that influences incontinence recovery 3–12 months after surgery.^15,16^ Importantly, our study did not find that older age remained a predictor of urinary function at 12 months. Rather, our results highlight that, although older men may experience worse outcomes in the weeks and months (5 weeks and 6 months post-RP) after surgery as compared to younger men, these differences diminished by 12 months post surgery. Counseling older patients about the projected timeline of continence recovery can help patients feel more prepared for post-surgical side effects and potentially reduce decisional regret.

To our knowledge, this is one of the first studies to examine the relationship between marital status and urinary function recovery post-RP. One small study among South Korean men found that marital intimacy had a positive influence on urinary symptoms^17^; however, most previous studies on marital status among prostate cancer patients have largely focused on mortality and other oncologic outcomes. Studies consistently report that being married at the time of RP is associated with decreased risk of all-cause and prostate cancer–specific mortality as well as higher quality of life.^7,8,18^ The higher baseline and 6-month post-RP scores that we observed in married men align with this trend. Importantly, we observed that the impact of marriage on post-RP urinary function was independent of age. This could be attributable to spouses promoting healthy behavior, such as doing Kegels or attending pelvic floor rehab, and is consistent well-documented positive influence of marriage and men’s health broadly.

There is limited previous literature on smoking and alcohol consumption with urinary function recovery. The studies to date are consistent with our finding that smoking is not associated with urinary function.^12^ However, in 3 of these studies, the definition of incontinence was limited to pads used per day rather than self-report.^19–21^ It is possible that men may experience declines in urinary function without requiring pads. In our study, we found no significant difference in urinary function between smokers and non-smokers at any time point, although ever-smokers had non-significantly lower mean urinary function scores at 6 months as compared to never-smokers.

In contrast to findings for smoking, studies based both in Japan and the UK observed that alcohol consumption was *inversely* associated with male urinary incontinence.^22,23^ This inverse pattern is consistent with our findings, although we did not observe statistically significant differences after RP. It is hypothesized that certain ingredients in alcohol can positively affect the contractile properties of the urethra and detrusor muscle leading to improved continence, though further research is needed to fully understand the underlying biological mechanisms.^22^ Alternatively, never-drinkers may be systematically different and potentially less healthy overall than people who consume alcohol. According to the “sick quitter” hypothesis, individuals who develop serious health conditions are more likely to cease drinking alcohol.^24^ For example, in one large Australian study, alcohol cessation was associated with 24–32 medical conditions examined,^24^ given that alcohol can be a contraindication for those with other pre-existing comorbidities. This bias could contribute to better health outcomes in alcohol consumers, consistent with our observations in this study.

### Sexual Function

Compared to urinary function, sexual function recovery was delayed and occurred to a lesser degree by 12 months post-RP. This finding is consistent with previous findings.^25,26^ Older age has been consistently identified as a prognostic factor for worse potency after RP.^27^ The post-surgical differences in recovery were largely driven by baseline differences in sexual function—older men started with lower sexual function and recovered to a worse endpoint as compared to their younger counterparts.

There is very limited and inconsistent evidence to date on marital status and sexual function.^17,25,28^ However, patient-reported spousal support has been associated with better erectile function both before and after RP^28^ and, in general, social support is associated with better surgical recovery and survival,^7,8,18^ which aligns with our findings. It is also possible that unmarried men underreport sexual function because they are less sexually active. Our results are also consistent with previous literature that suggests that smoking negatively impacts sexual function recovery.^12^ Smoking cessation improves penile blood flow in as little as 24–36 hours, which may explain our observation of lower sexual function recovery in men who smoke.^29^

Finally, there is extremely limited literature on alcohol consumption and sexual function recovery post-RP. Our study found an inverse relationship between frequency of alcohol consumption and baseline sexual function. By 12 months, this difference was no longer statistically significant; however, never-drinkers still had scores lower than monthly, weekly, or daily drinkers. Consistent with this finding, in a study among South Korean men, alcohol consumption was associated with better potency recovery with udenafil (a phosphodiesterase type 5 (PDE5) inhibitor used for erectile dysfunction) post-RP.^30^ More research is needed to understand the mechanism between alcohol use and sexual function; however, it is likely that a “sick quitter” bias could impact sexual function as well.^24^

### Clinical Relevance

Our results provide both patients and clinicians with realistic recovery trajectories based on patient characteristics. Men are more likely to experience decisional regret when they are not actively involved in the treatment–decision-making process and when treatment–related side effects are worse than anticipated.^4^ Our results can be used to inform accurate recovery expectations. Our findings also highlight the key role of baseline function on recovery post-RP, particularly for sexual function. This supports the idea that structured pre-surgical pre-rehabilitation could improve post-surgical recovery. Generally, smoking and alcohol cessation interventions help reduce postoperative complications.^10,11^ An expansion of such programs, particularly for smoking, could help improve the quality of life for men after RP. For example, pre-surgical smoking cessation programs could help improve sexual function recovery given that smokers recover to a lesser extent than non-smokers.

### Strengths and Limitations

Our study is limited by the use of self-report. Participants may underestimate habits viewed negatively (eg, smoking and alcohol use while diagnosed with prostate cancer) and overestimate traits viewed positively (eg, sexual competence). It is also possible that participants with objectively similar symptoms respond differently in scoring due to their subjective experience of urinary and sexual function. However, we expect this should be minimized given our use of a standardized and validated questionnaire. Moreover, this allows for our findings to more accurately represent the patient experience as compared to objective clinical measures that may or may not reflect this experience. Our measure of alcohol was limited to frequency, but did not account for the number of drinks consumed. This could have biased our measure of alcohol. In addition, 91% of the study population consisted of White men; therefore, we cannot necessarily generalize our findings to non-White men. Finally, our study was designed as a descriptive analysis and did not adjust for multiple hypothesis testing; results should be interpreted for exploratory purposes only. Despite these limitations, our study has several strengths. Our analysis allowed us to investigate the impact of important pre-surgical factors on treatment–related side effects that are relevant to patient quality of life at 3 different time points. Few existing studies have evaluated the impact of marital status, smoking status, and alcohol use on urinary and sexual function outcomes. Our study adds to the limited evidence to date. Moreover, the longitudinal nature of the PIE study provides comprehensive trajectories of recovery, which gives patients and providers more realistic and clinically relevant expectations of recovery.

## Conclusion

After RP, urinary function begins to recover at 6 months. However, recovery may be slower in older and unmarried patients. By 12 months post-RP, there were no differences in urinary function recovery across exposure groups and most men had recovered to baseline function. Sexual function did not recover to baseline levels for men in any group. At 12 months post-RP, sexual function scores were significantly lower for older, unmarried men, and ever-smokers.

## Supplementary Material

MMC1

Appendix A. Supporting information

Supplementary data associated with this article can be found in the online version at doi:10.1016/j.urology.2025.07.048.

## Figures and Tables

**Table 1.** Baseline characteristics of the PIE cohort.

Statistic	Urinary Function Population	Sexual Function Population
N	311	286
Age (y)*	60.93	60.58
	6.44	6.45
	61.00	61.00
Age (group)^†^		
Under 65	203	194
	65.27	67.83
65+	108	92
	34.73	32.17
Race^†^		
White	285	261
	91.64	91.26
Other	17	17
	5.47	5.94
* Missing*	9	8
	2.89	2.80
Highest attained education^†^		
Postgraduate	99	91
	31.83	31.82
College degree	75	67
	24.12	23.43
Some college	84	79
	27.01	27.62
High school or less	46	43
	14.79	15.03
* Missing*	7	6
	2.25	2.10
Marital status^†^		
Married or living with someone	266	245
	85.53	85.66
Not otherwise married	38	36
	12.22	12.59
* Missing*	7	5
	2.25	1.75
Alcohol consumption frequency^†^		
Never	55	53
	17.68	18.53
Monthly	68	62
	21.86	21.68
Weekly	109	101
	35.05	35.31
Daily	75	68
	24.12	23.78
* Missing*	4	2
	1.29	0.70
Smoking status^†^		
Never smoker	182	173
	58.52	60.49
Ever smoker	122	107
	39.23	37.41
* Missing*	7	6
	2.25	2.10

PIE, Prostatectomy, Incontinence, and Erectile function.

All variables are measured from baseline (ie, pre-treatment) unless otherwise specified.

* Data reported: mean (top); standard deviation (middle); median (bottom).

† Data reported: frequency (top); column percentage (bottom).

## Data Availability

The dataset supporting the conclusion of this article is available upon request.
