# Supplementary material for: Impact of Age, Marital Status, Smoking, and Alcohol Consumption on Urinary and Sexual Function in Prostate Cancer Patients Treated With Radical Prostatectomy: A Prospective Cohort Study
Source: Urology. Author manuscript; Available in PMC 2026 Jan 21. (PMC12823265; doi:10.1016/j.urology.2025.07.048)
Supplement: MMC1 [file NIHMS2120970-supplement-MMC1.pdf]

Supplementary Table 1: Urinary Function by Patient Characteristics (unadjusted)					
**Population**		**Baseline (Pre-Tx)**	**5 Weeks Post-Tx**	**6 Months Post-Tx**	**12 Months Post-Tx**
**Age Group**					
**Under-65 (N = 203)**	Mean	93.54	58.43	82.99	86.53
	Standard Deviation	10.40	18.32	15.07	13.46
	Median	100.00	56.80	83.40	88.40
**65+ (N = 108)**	Mean	92.04	54.10	78.90	84.55
	Standard Deviation	11.90	17.28	17.90	15.91
	Median	100.00	53.40	81.80	88.40
**Marital Status**					
**Married or living with someone (N = 266)**	Mean	93.61	57.11	82.50	86.45
	Standard Deviation	10.29	17.57	15.60	13.58
	Median	100.00	55.00	83.40	88.40
**Not otherwise married (N = 38)**	Mean	88.04	54.16	73.82	80.33
	Standard Deviation	14.46	20.44	18.26	18.98
	Median	95.00	56.80	73.40	85.90
**Smoking Status**					
**Never Smokers (N = 182)**	Mean	92.84	56.57	82.21	86.07
	Standard Deviation	11.24	18.64	16.24	14.19
	Median	100.00	53.40	83.40	88.40
**Ever Smokers (N = 122)**	Mean	93.32	57.29	80.10	85.30
	Standard Deviation	10.57	17.16	16.29	14.88
	Median	100.00	56.70	82.60	88.40
**Alcohol Consumption**					
**Never (N = 55)**	Mean	90.22	54.90	79.63	84.69
	Standard Deviation	12.75	20.02	18.40	15.36
	Median	95.00	53.20	81.80	88.40
**Monthly (N = 68)**	Mean	92.78	54.87	79.69	84.96
	Standard Deviation	11.15	18.01	17.17	15.54
	Median	100.00	53.20	78.40	88.40
**Weekly (N = 109)**	Mean	94.83	56.88	82.63	86.82
	Standard Deviation	10.16	19.08	15.25	14.16
	Median	100.00	58.40	83.40	88.40
**Daily (N = 75)**	Mean	92.28	60.16	82.95	85.61
	Standard Deviation	10.32	14.68	14.69	13.17
	Median	100.00	60.00	85.50	88.40

Supplementary Table 2: Urinary Function by Patient Characteristics (Age adjusted)					
**Population**		**Baseline (Pre-Tx)**	**5 Weeks Post-Tx**	**6 Months Post-Tx**	**12 Months Post-Tx**
**Marital Status**					
**Married or living with someone (N = 266)**	Mean	93.65	57.19	82.59	86.51
	Standard Deviation	0.66	1.09	0.96	0.87
	95% CI	92.34 – 94.95	55.04 – 59.33	80.70 – 84.48	84.80 – 88.23
**Not otherwise married (N = 38)**	Mean	87.74	53.62	73.14	79.88
	Standard Deviation	1.76	2.89	2.55	2.31
	95% CI	84.29 – 91.20	47.92 – 59.31	68.13 – 78.15	75.33 – 84.42
**Smoking Status**					
**Never Smokers (N = 182)**	Mean	92.50	55.87	81.58	85.61
	Standard Deviation	0.82	1.34	1.21	1.08
	95% CI	90.89 – 94.12	53.22 – 58.51	79.20 – 83.96	83.49 – 87.74
**Ever Smokers (N = 122)**	Mean	93.82	58.34	81.04	85.99
	Standard Deviation	1.01	1.65	1.49	1.33
	95% CI	91.83 – 95.81	55.09 – 61.60	78.11 – 83.97	83.36 – 88.61
**Alcohol Consumption**					
**Never (N = 55)**	Mean	90.35	55.23	79.97	84.90
	Standard Deviation	1.47	2.41	2.15	1.94
	95% CI	87.46 – 93.24	50.49 – 59.97	75.74 – 84.20	81.08 – 88.72
**Monthly (N = 68)**	Mean	92.81	54.93	79.75	85.01
	Standard Deviation	1.32	2.16	1.93	1.74
	95% CI	90.21 – 95.41	50.68 – 59.19	75.95 – 83.55	81.58 – 88.44
**Weekly (N = 109)**	Mean	94.71	56.59	82.33	86.63
	Standard Deviation	1.04	1.71	1.53	1.38
	95% CI	92.66 – 96.77	53.22 – 59.96	79.33 – 85.34	83.91 – 89.34
**Daily (N = 75)**	Mean	92.32	60.28	83.07	85.69
	Standard Deviation	1.26	2.06	1.84	1.66
	95% CI	89.85 – 94.80	56.23 – 64.34	79.45 – 86.69	82.42 – 88.95

Supplementary Table 3: Sexual Function by Patient Characteristics (unadjusted)					
**Population**		**Baseline (Pre-Tx)**	**5 Weeks Post-Tx**	**6 Months Post-Tx**	**12 Months Post-Tx**
**Age Group**					
**Under-65 (N = 194)**	Mean	62.53	24.06	29.97	35.43
	Standard Deviation	22.98	23.12	22.53	24.48
	Median	69.44	16.67	24.56	32.44
**65+ (N = 92)**	Mean	46.11	13.06	20.63	22.94
	Standard Deviation	24.43	15.34	1.909	21.77
	Median	51.89	5.90	15.20	14.33
**Marital Status**					
**Married or living with someone (N = 245)**	Mean	57.79	20.68	27.47	32.26
	Standard Deviation	23.87	21.47	21.76	24.35
	Median	62.50	13.89	20.33	26.89
**Not otherwise married (N = 36)**	Mean	53.06	17.56	21.52	23.27
	Standard Deviation	29.84	19.76	20.13	21.30
	Median	66.15	9.72	21.28	24.06
**Smoking Status**					
**Never Smokers (N = 173)**	Mean	61.65	22.58	29.72	34.76
	Standard Deviation	23.33	22.80	21.52	24.37
	Median	68.75	13.89	24.11	32.44
**Ever Smokers (N = 107)**	Mean	50.13	16.75	21.97	25.46
	Standard Deviation	25.49	19.10	21.83	23.39
	Median	54.67	11.11	13.89	19.44
**Alcohol Consumption**					
**Never (N = 53)**	Mean	48.12	16.35	23.25	26.32
	Standard Deviation	27.18	16.90	1.986	24.62
	Median	49.11	12.50	17.56	19.44
**Monthly (N = 62)**	Mean	56.61	18.81	22.94	26.92
	Standard Deviation	23.78	22.94	20.67	21.35
	Median	62.50	10.61	18.50	21.56
**Weekly (N = 101)**	Mean	60.02	21.29	28.16	34.06
	Standard Deviation	23.70	21.27	21.31	24.27
	Median	64.63	13.89	22.88	29.67
**Daily (N = 68)**	Mean	60.44	23.66	30.86	34.67
	Standard Deviation	23.73	23.61	24.40	25.74
	Median	63.89	13.89	24.11	31.06

Supplementary Table 4: Sexual Function by Patient Characteristics (Age adjusted)					
**Population**		**Baseline (Pre-Tx)**	**5 Weeks Post-Tx**	**6 Months Post-Tx**	**12 Months Post-Tx**
**Marital Status**					
**Married or living with someone (N = 245)**	Mean	57.79	20.68	27.47	32.26
	Standard Deviation	23.87	21.47	21.76	24.35
	95% CI	62.50	13.89	20.33	26.89
**Not otherwise married (N = 36)**	Mean	53.06	17.56	21.52	23.27
	Standard Deviation	29.84	19.76	20.13	21.30
	95% CI	66.15	9.72	21.28	24.06
**Smoking Status**					
**Never Smokers (N = 173)**	Mean	59.58	21.52	28.56	33.18
	Standard Deviation	1.71	1.61	1.62	1.76
	95% CI	56.21 – 62.95	18.35 – 24.70	25.38 – 31.75	29.71 – 36.66
**Ever Smokers (N = 107)**	Mean	53.48	18.46	23.85	28.00
	Standard Deviation	2.19	2.07	2.07	2.26
	95% CI	49.16 – 57.80	14.39 – 22.54	19.76 – 27.93	23.54 – 32.45
**Alcohol Consumption**					
**Never (N = 53)**	Mean	49.66	17.15	24.16	27.51
	Standard Deviation	3.03	2.87	2.86	3.12
	95% CI	43.69 – 55.63	11.51 – 22.79	18.53 – 29.80	21.36 – 33.66
**Monthly (N = 62)**	Mean	56.04	18.51	22.61	26.48
	Standard Deviation	2.80	2.65	2.64	2.88
	95% CI	50.54 – 61.55	13.31 – 23.72	17.40 – 27.81	20.81 – 32.16
**Weekly (N = 101)**	Mean	58.94	20.72	27.52	33.22
	Standard Deviation	2.20	2.08	2.07	2.26
	95% CI	54.61 – 63.26	16.64 – 24.81	23.44 – 31.60	28.77 – 37.67
**Daily (N = 68)**	Mean	61.37	24.14	31.41	35.39
	Standard Deviation	2.67	2.53	2.53	2.75
	95% CI	56.11 – 66.63	19.17 – 29.12	26.44 – 36.38	29.97 – 40.81
